# Severe asthma in adolescence - or how the low self-esteem obstacles the therapy

**DOI:** 10.1186/2045-7022-5-S2-P20

**Published:** 2015-03-23

**Authors:** Guergana Petrova, Sylvia Shopova, Dimitrinka Miteva, Snezhina Lazova, Penka Perenovska

**Affiliations:** 1 UH "Alexandrovska", Pediatric clinic, Sofia, Bulgaria

## Background

Adolescent with any chronic disease face rebellious stage of puberty aggravated with the condition they have usually requiring strict food and behavioral regimen. Sometimes during this period a seemingly “well-controlled” asthma turns out to be “uncontrolled” despite the increase in therapy and thus classifying it as a severe asthma as defined as ERS/ATS guidelines.

## Clinical case

We present a case of 17-year old girl with bronchial asthma, hospitalized in the clinic multiple times, despite high dose of combined corticosteroids as a controller medication. The child starts to show protest behavior towards therapy, that’s modifying in the course of psychological maturation – denial of the medicines, unhealthy and hazardous life styles. At the age of 16-years depression was diagnosed, and was pharmacological and psychological therapy. This case is presented with aim to show specific for most of the asthmatic patients’ negative self-estimation for their quality of life and how it requires complex theurapetical approach.

## Conclusion

A modern team-work approach with respiratory/allergy specialist and psychology/psychiatry specialist sometimes is the best way to manage teenagers with difficult to treat asthma.

## Consent

Written informed consent was obtained from the patient for publication of this abstract and any accompanying images. A copy of the written consent is available for review by the Editor of this journal.

